# Epitope spreading to citrullinated antigens in mouse models of autoimmune arthritis and demyelination

**DOI:** 10.1186/ar2523

**Published:** 2008-09-30

**Authors:** Brian A Kidd, Peggy P Ho, Orr Sharpe, Xiaoyan Zhao, Beren H Tomooka, Jennifer L Kanter, Lawrence Steinman, William H Robinson

**Affiliations:** 1 Department of Medicine, Division of Immunology and Rheumatology, CCSR 4135, 269 Campus Dr., Stanford University School of Medicine, Stanford, CA, USA; 2 GRECC, Palo Alto VA Health Care System, 3801 Miranda Ave., Palo Alto, CA, USA; 3 University of Washington, 1705 NE Pacific St., Seattle, WA, USA; 4 Department of Neurology and Neurological Sciences, Stanford University School of Medicine, Beckman B-002, 279 Campus Dr., Stanford, CA, USA

## Abstract

**Introduction:**

Anti-citrullinated protein antibodies have a diagnostic role in rheumatoid arthritis (RA); however, little is known about their origins and contribution to pathogenesis. Citrullination is the post-translational conversion of arginine to citrulline by peptidyl arginine deiminase, and increased citrullination of proteins is observed in the joint tissue in RA and in brain tissue in multiple sclerosis (MS).

**Methods:**

We applied synovial and myelin protein arrays to examine epitope spreading of B cell responses to citrullinated epitopes in both the collagen-induced arthritis (CIA) model for RA and the experimental autoimmune encephalomyelitis (EAE) model for MS. Synovial and myelin protein arrays contain a spectrum of proteins and peptides, including native and citrullinated forms, representing candidate autoantigens in RA and MS, respectively. We applied these arrays to characterise the specificity of autoantibodies in serial serum samples derived from mice with acute and chronic stages of CIA and EAE.

**Results:**

In samples from pre-disease CIA and acute-disease EAE, we observed autoantibody targeting of the immunising antigen and responses to a limited set of citrullinated epitopes. Over the course of diseases, the autoantibody responses expanded to target multiple citrullinated epitopes in both CIA and EAE. Using immunoblotting and mass spectrometry analysis, we identified citrullination of multiple polypeptides in CIA joint and EAE brain tissue that have not previously been described as citrullinated.

**Conclusions:**

Our results suggest that anti-citrulline antibody responses develop in the early stages of CIA and EAE, and that autoimmune inflammation results in citrullination of joint proteins in CIA and brain proteins in EAE, thereby creating neoantigens that become additional targets in epitope spreading of autoimmune responses.

## Introduction

Post-translational modifications can create neo-antigens that become targets of autoimmune responses [1,2]. A post-translational modification of importance in rheumatoid arthritis (RA) is the conversion of peptidylarginine to peptidylcitrulline by peptidyl arginine deiminase (PAD) [3,4]. This enzymatic modification is termed citrullination, and detection of antibodies targeting citrullinated epitopes has become a key diagnostic marker for the diagnosis of RA, with a sensitivity of about 60% and a specificity of 95% [5-10]. Nevertheless, the mechanisms by which anti-citrullinated protein antibody (ACPA) responses develop remain poorly understood. In this study, we used protein microarrays to characterise ACPA responses in rodent models of RA and multiple sclerosis (MS).

In RA, ACPA responses are currently assayed in clinical laboratories by detection of antibodies targeting cyclic citrullinated peptides (CCPs) derived from filaggrin [7]. These CCPs probably represent molecular mimics of the true citrullinated autoantigens in RA synovium. In RA and other inflammatory arthritides, multiple joint proteins become citrullinated [10-12]. Recent evidence suggests that cigarette smoking induces citrullination of lung proteins, and in patients possessing the shared epitope of human leucocyte antigen (HLA) DR, smoking is associated with about a 20-fold increased risk of developing anti-CCP antibody-positive RA [13]. Further, Kuhn and colleagues showed that a monoclonal antibody specific for citrullinated fibrinogen exacerbated tissue injury in rodent models of arthritis [14], although it remains possible that this non-affinity matured IgM antibody might cross-react with a native cartilage component as described for anti-citrulline IgG responses in RA [15]. Thus, it is possible that cigarette smoking represents an environmental trigger that induces anti-citrulline antibody responses in genetically susceptible individuals and thereby contributes to the development of RA [13].

Alterations in the citrullination of myelin proteins and autoimmune targeting of citrullinated myelin proteins have been observed in MS. Myelin basic protein (MBP) is partially citrullinated (C8 isoform) in normal brain tissue, although there is a significant increase in the relative amount of this partially citrullinated form in MS brain tissue [15]. An extensively citrullinated form of MBP is associated with Marburg encephalitis, a fulminant autoimmune demyelinating disease [16]. There have also been reports of anti-citrullinated-MBP T cell reactivity in MS [17], as well as evidence that citrullinated MBP can serve as an autoantigen in experimental autoimmune encephalomyelitis (EAE) [18].

In this study we applied synovial and myelin protein arrays to investigate the development and evolution of ACPA responses in rodent models of RA and MS, with the objective of gaining further insights into the aetiology, evolution and potential pathogenic role of such responses in human RA and MS. We demonstrate targeting of a limited number of citrullinated polypeptides in pre-disease samples derived from mice with collagen-induced arthritis (CIA) and early disease samples derived from mice with EAE, and expansion of responses to target multiple citrullinated molecules in established and long-standing disease. Mass spectroscopy analysis identified citrulline-modifications in multiple proteins in inflamed CIA joint and EAE brain tissue. Our results suggest that citrullination of synovial proteins in CIA and brain proteins in EAE generate neoantigens capable of provoking anti-citrulline antibody responses.

## Materials and methods

### CIA and EAE

All animal experiments were conducted under approval from the Stanford University Institutional Animal Care and Use Committee. CIA was induced in DBA1/J mice by an intradermal injection of collagen type II (CII) (100 μg/mouse) emulsified in complete Freund's adjuvant (CFA) containing 5 mg/mL heat-killed *Mycobacterium tuberculosis *H37Ra (Difco Laboratories, Detroit, MI, USA), followed by boosting with CII (100 μg/mouse) emulsified in incomplete Freund's adjuvant on day 21. Animals were scored as previously described [21]. Serum was collected from CIA mice at the pre-boost (day 21), early arthritis (day 31) and chronic (day 55) stages of the disease. In addition, serum was collected from control mice that were matched in age and strain to the diseased mice described above.

For the induction of EAE, SJL/J mice were given a subcutaneous injection of proteolipid protein (PLP) amino acids 139–151 (PLP p139–151) (100 μg/mouse) emulsified in CFA [20]. Mice were scored daily for EAE as previously described [22], and mouse serum was collected at time points that corresponded to specific stages of disease or treatment. In SJL mice, serum was collected at the acute (day 13), intermediate (day 28) and chronic (day 67) phases of disease.

### Peptides and proteins

Tissue-specific antigen microarrays were generated to characterise autoantibody responses in CIA and EAE. 'Synovial microarrays' were used to profile the autoantibody responses in CIA [19]. Synovial microarrays contained 253 antigens, including 213 peptides, 38 proteins and two nucleic acids, which largely represented components of synovial joints along with other antigens of interest and controls. 'Myelin microarrays' were used to profile the autoantibody response in EAE [20]. Myelin arrays contained 406 antigens, including 375 peptides, 28 proteins and three nucleic acids, which largely represented components of the myelin sheath along with other antigens of interest and controls. A detailed list of the peptides and proteins included on each array is provided (additional files 1 and 2).

The synovial and myelin microarrays contained mouse and/or human versions of the proteins and peptides, depending on their availability from the collaborators that provided these antigens. Their origins are indicated in additional files 1 and 2. Many of the polypeptide sequences are highly conserved between species, and thus most protein and peptides provide utility for screening for autoantibody reactivity across species.

### Array production and assay

**Figure 1:**
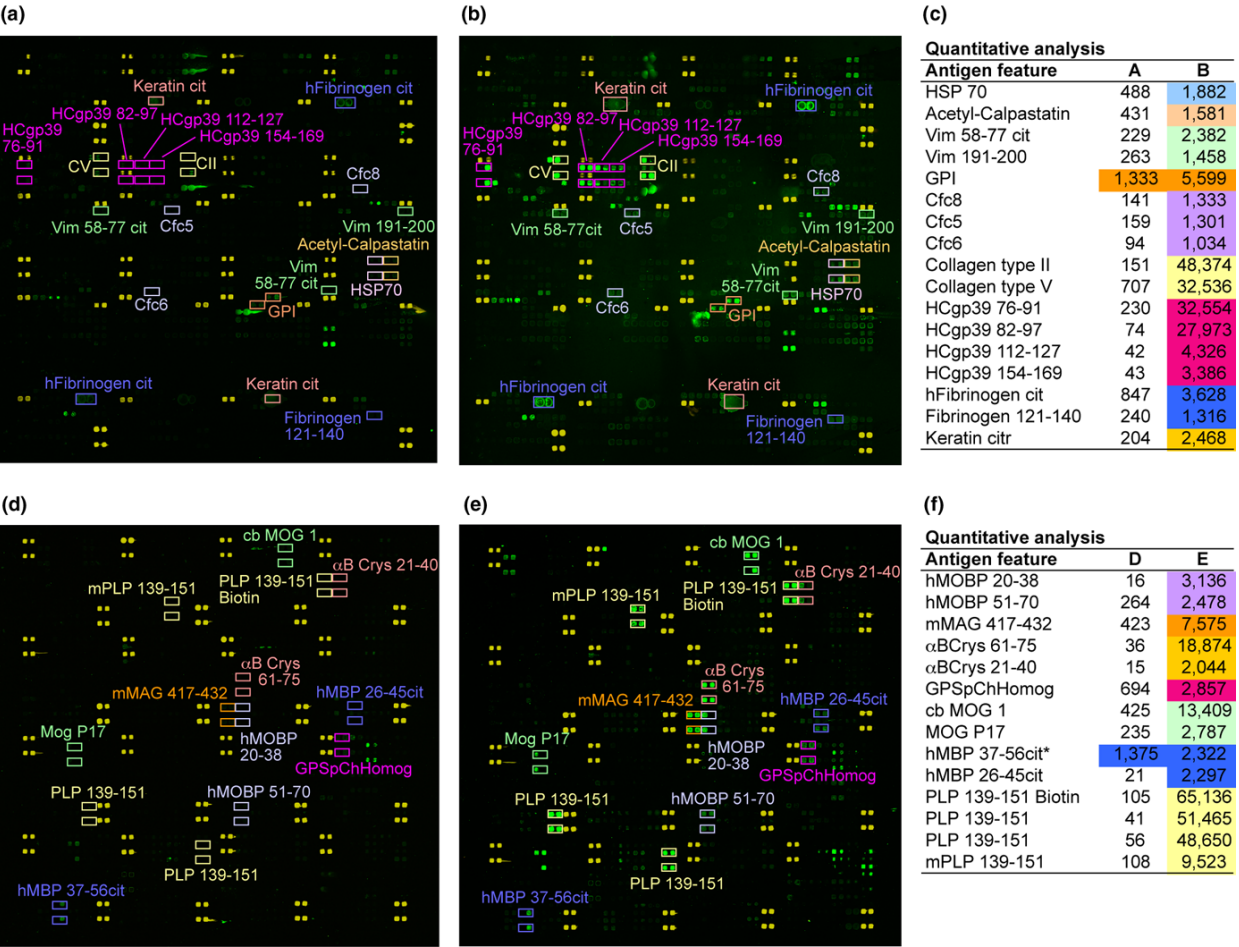
**Characterisation of antibody responses in collagen-induced arthritis (CIA) and experimental autoimmune encephalomyelitis (EAE) using myelin and synovial antigen arrays**. (a, b) Synovial and (d, e) myelin arrays were produced by printing synovial and myelin peptides and proteins in ordered arrays on the surface of microscope slides. The arrays were probed with 1:150 dilutions of (a, d) normal, (b) CIA and (e) EAE sera followed by a cyanine 3 labelled anti-mouse IgG/M secondary antibody. Scanned images of the slides are presented in false colour representation. Yellow features represent marker spots to orient the array. Green features represent serum antibody reactivity. Reactive features are highlighted and quantitative values are presented in panels c and f.

Synovial and myelin antigen arrays were produced using a robotic arrayer. The arrayer printed peptides and proteins representing candidate antigens in RA and MS on the surface of SuperEpoxy 2 Protein Substrates (Telechem International, Sunnyvale, CA, USA) [20,23]. Each array contained four to 12 replicates of each peptide or protein. Arrays were blocked overnight at 4°C in PBS containing 3% FCS and 0.5% Tween-20. After blocking, arrays were incubated with 1:150 dilutions of mouse serum in buffer (PBS plus 3% FCS) for one hour at 4°C. After incubation, arrays were washed and incubated with 1:3500 dilutions of cyanine 3 dye conjugated goat anti-mouse IgG/M (Jackson Immunoresearch, West Grove, PA, USA) for 45 minutes at 4°C. Following the second incubation, arrays were washed, spun dry and scanned with a GenePix 4000 B scanner (Axon Instruments, Union City, CA, USA) to generate digital images (Figure 1). Detailed protocols were published previously [20,24] and are available online [25].

### Data analysis

**Figure 2:**
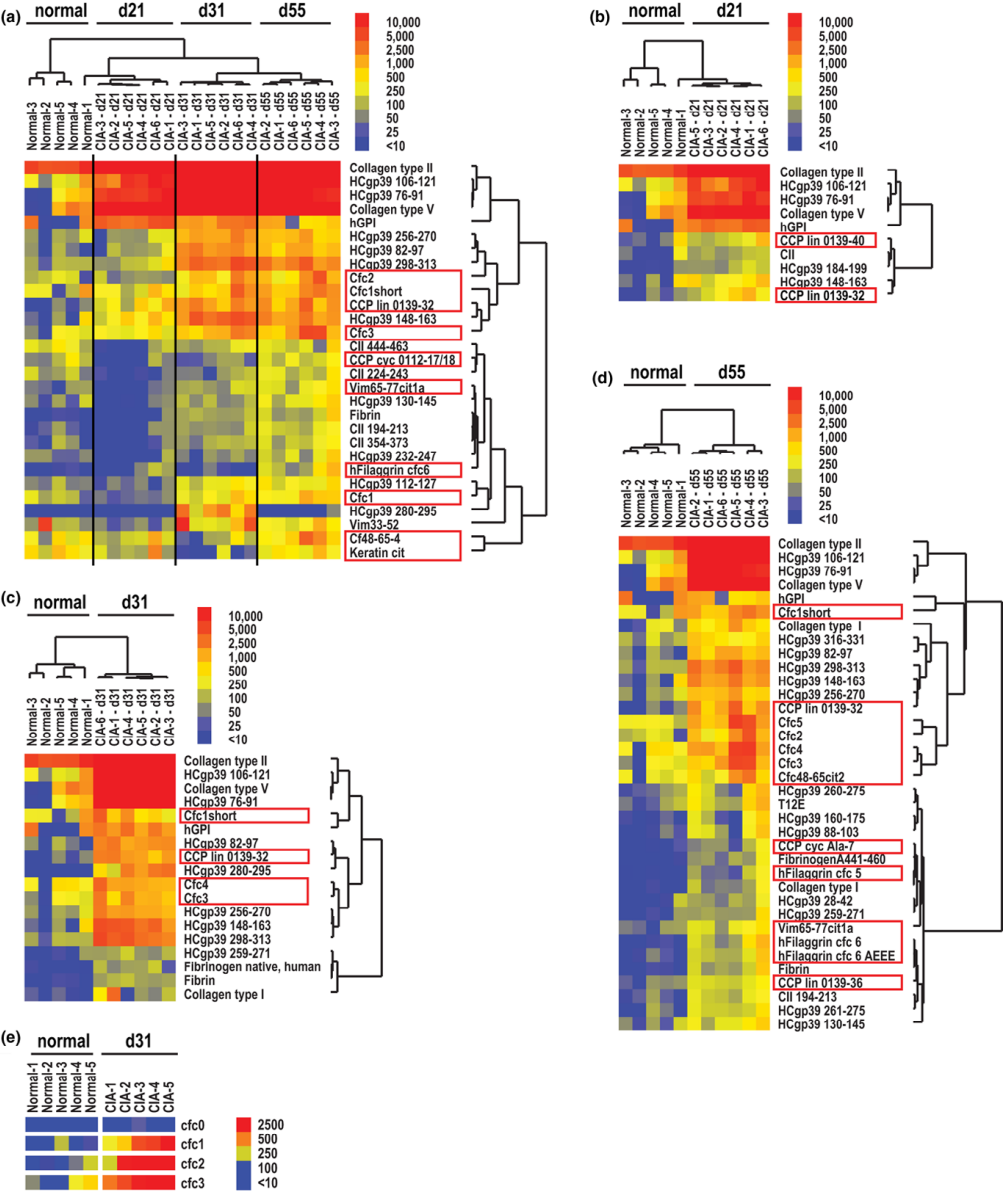
**Synovial arrays demonstrate epitope spreading of autoantibodies targeting native and citrullinated molecules in collagen-induced arthritis (CIA)**. CIA was induced in DBA1/J mice using CII emulsified in complete Freund's adjuvant. The mice developed clinical CIA about one week after boosting (days 26 to 31). Serum was obtained on day 21, 31 and 55, representing pre-arthritis, early arthritis and late-stage arthritis, respectively. Significance Analysis of Microarrays (SAM) identified antigen features (displayed to the right of the images) with statistically significant differences in array reactivity, with citrullinated peptides and proteins denoted by red boxes. A hierarchical cluster algorithm based on a pairwise similarity function was used to order SAM-identified antigens and mice into relationships. The relationships are presented in tree-dendograms where branch lengths represent the degree of similarity between mice or antigen features. Blue represents lack of reactivity, yellow low and red positive reactivity. (a) Multiclass SAM followed by cluster analysis demonstrating statistical differences in antibody reactivity differences between healthy (normal) mice and mice induced for CIA at the pre-boost (day 21), early arthritis (day 31) and late-stage arthritis timepoints. Two-class SAM followed by cluster analysis presenting differences in antibody reactivity between healthy (normal) and mice immunised for CIA at the (b) pre-boost, (c) early arthritis and the (d) late stage arthritis timepoints. (e) Direct comparison of Cfc0, native filaggrin (306–324) epitope and variants of citrullinated filaggrin Cfc1-Cfc3.

GenePix Pro 5.0 software (Axon Instruments, Union City, CA, USA) was used to determine the median pixel intensity for each feature on the array. Each feature contained four to 12 replicates so the final intensity (antigen reactivity) of a unique feature was the median of these replicates. Median intensity values were processed for statistical analysis by setting all values less than 10 to 10, normalising the intensities by 300, and then applying a log base two transformation to every normalised value. Processed values with no variation between arrays were eliminated. Statistical analysis was performed using Significance Analysis of Microarrays (SAM) (Dr. Robert Tibshirani, Stanford University, Stanford, CA, USA) [26,27] to identify antibodies with statistical differences in antigen reactivity between groups. Results were selected based on a false discovery rate of less than 0.05 combined with a numerator threshold of 2.5 (Figures 2b,c,d), and SAM results were clustered and displayed using Cluster 2.12 [28] and TreeView 1.60 [28] software (Lawrence Berkeley National Lab and the University of California Berkeley, Berkeley, CA, USA)

### Immunoblot analysis

CIA synovial tissue and EAE brain tissue were minced and the protein contents extracted with tissue protein extraction buffer (T-PER, Pierce Biotechnology, Rockford, IL). The amount of protein loaded in each lane was controlled for based on bicinchoninic acid (BCA) quantification of protein concentrations in the lysates, and 50 mg of total protein was loaded in each lane. Of each protein lysate, 50 mg was diluted in 150 μL of isoelectric focusing (IEF) buffer (8 M urea, 20 mM dithiothreitol (DTT), 2% w/v Chaps, 0.2% Biolytes, 2 M thiourea and bromophenol blue), and the lysates separated with 11 cm Ready-Strip IPG strips pH 3–10 (Bio-Rad, Hercules, CA) with a Protean IEF Cell (Bio-Rad, Hercules, CA). Precast Criterion Tris-HCl gels (4 to 20% linear gradient, Bio-Rad, Hercules, CA) were used to perform second-dimension electrophoresis, and separated proteins blotted onto nitrocellulose membranes. After blocking with 3% BSA in PBS, immunoblotting with anti-modified citrulline was performed with an anti-citrulline detection kit (Upstate, Chicago, IL) according to the manufacturer's instructions. Bound antibodies were detected with horse radish peroxidase (HRP)-conjugated anti-mouse IgM/G (Jackson Immunoresearch, West Grove, PA, USA) using a SuperSignal kit (Pierce Biochemicals, Rockford, IL, USA) and chemiluminescence was imaged with FluorChem imaging system (Alpha Innotech, San Leandro, CA, USA).

### Mass spectrometry analysis

Protein spots were excised from the gel and treated with trypsin overnight at 37°C. The tryptic peptides were resolved by HPLC using a Zorbax 300SB-C18 nanocolumn packed with 3.5 μm particles (Agilent Technologies, Santa Clara, CA, USA) and eluted at 300 nL/minute with a 60-minute linear gradient from 0 to 95% acetonitrile containing 0.1% formic acid. Separated peptides were electrosprayed into an ion trap mass spectrometer (XCT Plus, Agilent Technologies, Santa Clara, CA, USA). Proteins were identified based on raw MS/MS data compared with a SwissProt database using Mascot (Matrix Science, Boston, MA, USA) with more than three valid peptide hits.

### ELISA

Anti-CCP ELISA (Axis-Shield, Dundee, Scotland, USA) was performed on mouse serum collected from control and CIA mice. The protocol was adapted from the manufacturer's suggested procedure, with substitution of 1:150 dilutions of mouse serum and a goat-anti-mouse IgG/M specific secondary antibody (Jackson Immunoresearch, West Grove, PA, USA). P values are provided for comparison of mean reactivity between control and CIA mice using the Mann-Whitney test.

## Results

### Synovial and myelin antigen arrays for characterising autoantibody responses

We used protein microarrays to determine the specific autoantibody response in two separate murine models for autoimmunity, CIA and EAE. Individual peptide or protein antigens were printed in four to 12 replicates (identical features) on each array, and median values for all features representing an individual antigen were used for data analysis. The 253 antigen synovial arrays contained a spectrum candidate autoantigen in RA, including epitopes from human cartilage glycoprotein 39 (HCgp39), filaggrin, calpastatin, vimentin, keratin, fibrinogen and multiple collagen types [5,6,9,29-34]. Serum from healthy DBA1/J mice lacked autoantibodies against epitopes included on the synovial arrays (Figure 1a), while serum from DBA1/J mice immunised with CII demonstrated autoantibodies targeting CII and multiple other RA relevant epitopes represented on the synovial arrays (Figure 1b).

The 406 antigen myelin arrays contained whole proteins and synthetic peptides that represent candidate autoantigens in MS [35-37]. Serum from healthy SJL mice showed no reactivity against myelin antigens (Figure 1d), whereas serum from SJL mice with EAE showed autoreactive antibodies against multiple myelin epitopes (Figure 1e). Arrays probed with EAE serum demonstrated high reactivity against the epitope used for immunisation (PLP 139–151), along with strong autoantibody reactivity against epitopes derived from MBP, myelin oligodendrocyte glycoprotein (MOG), myelin-associated glycoprotein, myelin-associated oligodendrocyte basic protein and αB-crystallin.

### Epitope spreading of autoantibody responses in CIA

We probed synovial arrays with serial serum samples to characterise the pattern of epitope spreading in CIA. For these experiments, the 253 antigen synovial arrays were probed with 1:150 dilutions of CIA sera collected at the indicated serial timepoints. SAM was applied to determine the antigens with statistical differences in array reactivity between the timepoints. For example, multiclass SAM analysis was performed and SAM identified the specific set of antigens (from the 253 represented on the arrays) that demonstrated significant differences between health mice, and mice induced for CIA at 21 days (pre-boost), 31 days (early arthritis) and 55 days (chronic arthritis) (Figure 2a). Two-class SAM analyses were also performed to determine the differences in autoantibody reactivity between healthy mice and mice at each individual timepoint (Figures 2b,c,d). Figure 2 presents the development and evolution of autoantibody responses in CIA.

Array analysis demonstrated successive increases in autoantibody reactivity as mice immunised for CIA progressed from the pre-boosting phase (day 21), to early arthritis (day 31), to chronic arthritis (day 55) (Figure 2a). Twenty-one days after immunisation (pre-boosting), immunised mice possessed autoantibodies that reacted against a small panel of epitopes derived from CII (the autoantigen used for immunisation), type V collagen, HCgp39 and glucose-6-phosphate isomerase (GPI) (Figure 2b). In addition, before boosting on day 21, mice exhibited a low-level response against two citrullinated filaggrin peptides. After development of clinical arthritis on about day 28, mice exhibited autoantibodies targeting multiple additional native and citrullinated epitopes (Figure 2c). These responses significantly expand in chronic CIA (day 55) to target a broad spectrum of native and citrullinated molecules (Figure 2d).

Non-citrullinated native variants of all citrullinated peptides were included on the microarrays and for the vast majority of the reactive citrullinated peptides the native variants were non-reactive. Figure 2e presents an example of such results, with citrulline-substituted variants (Cfc1, Cfc2 and Cfc3) of a single filaggrin peptide (Cfc0) demonstrating significant reactivity, although the native variant (Cfc0) was non-reactive.

Anti-CII antibody levels were identified by SAM as being significantly different between sera derived from mice induced for CIA (Figure 2) (day 21 median anti-CII antibody reactivity = 65,054 digital fluorescence units [dfu]; day 31 = 63,161 dfu; day 55 = 64,450 dfu) as compared with naïve DBA/1 mice (5542 dfu). We frequently observe fluorescence at CII features in sera derived from naïve mice, and it is possible that some non-specific binding occurs to CII protein (unpublished results). Nevertheless, the colour scale used to display array reactivities masks the statistically significant and large magnitude (10+ fold) differences in anti-CII antibody reactivity detected in CIA-immunised mice as compared with naïve DBA/1 mice.

### Epitope spreading of autoantibody responses in EAE

**Figure 3:**
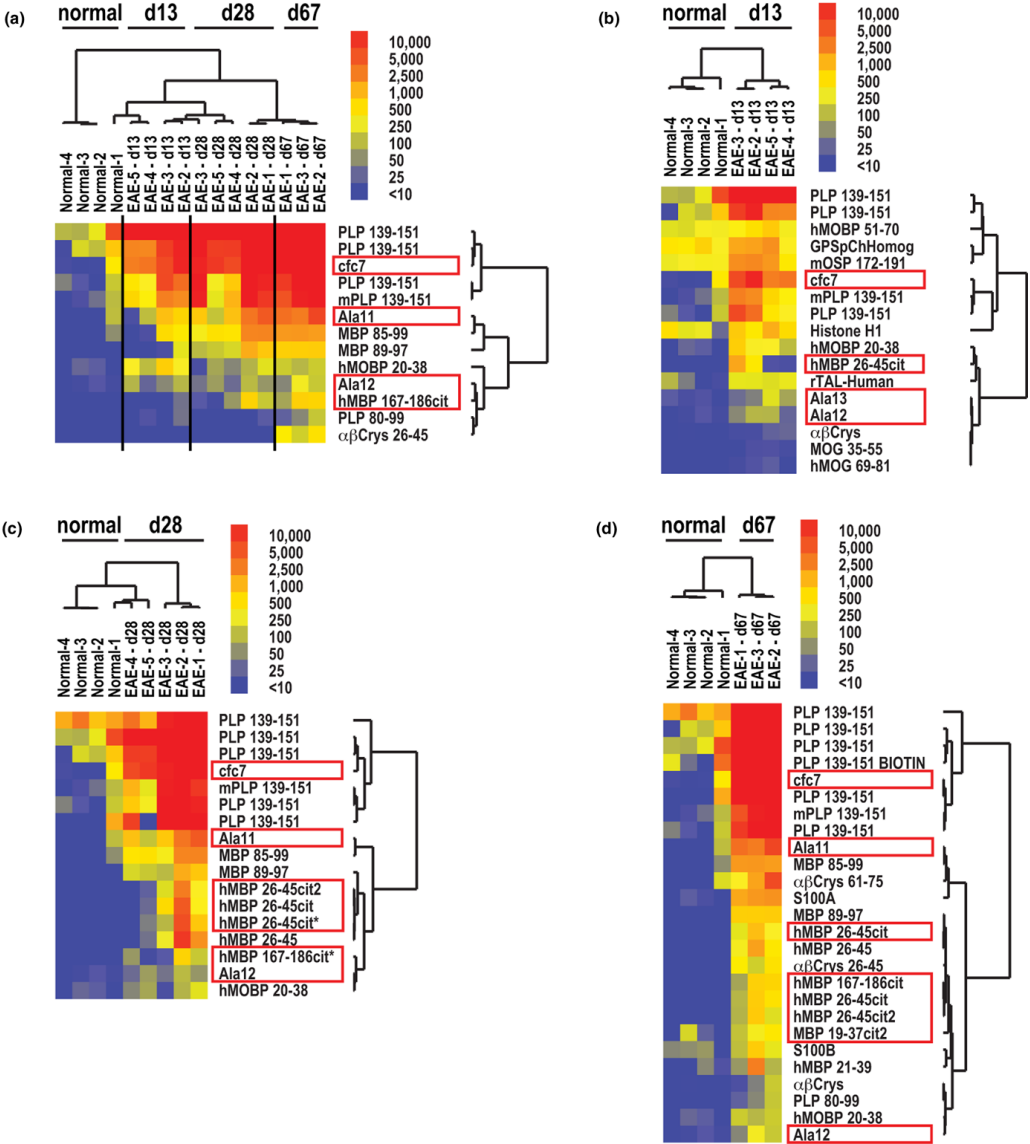
**Myelin arrays demonstrate epitope spreading of autoantibody responses to target native and citrullinated epitopes in experimental autoimmune encephalomyelitis (EAE)**. EAE was induced in SJL mice using proteolipid protein (PLP) (p139–151) emulsified in complete Freund's adjuvant. Mice developed clinical EAE on days 10 to 14. On days 13, 28 and 67 serum was obtained and myelin array analysis performed. Significance Analysis of Microarrays (SAM) was used to identify antibodies with statistical differences in reactivity between the timepoints and the results displayed as a heatmap. Red boxes denote citrullinated peptides and proteins. (a) Multiclass SAM followed by cluster analysis demonstrating statistical differences in antibody reactivity differences between healthy (normal) mice and mice with acute EAE (day 13), mid-stage EAE (day 28) and long-standing EAE (Day 67). Two-class SAM followed by cluster analysis presenting differences in antibody reactivity between healthy (normal) and mice with (b) acute, (c) mid-stage and (d) long-standing EAE.

We used 406 antigen myelin proteome arrays to examine the pattern of epitope spreading of the autoantibody response in EAE. Epitope spreading correlated with disease progression from an acute onset at day 10 to 14 to a chronic phase at day 28 and 67 (Figure 3a). High-titre autoantibody targeting of PLP139–151, the inducing antigen, was observed at the acute onset in SJL mice with EAE (Figure 3b). By day 67, this response had spread to additional epitopes on PLP, as well as to epitopes on other myelin proteins including MOG (MOG 27–50 and MOG 79–90), MBP (MBP 21–39, MBP 26–45, MBP 85–99 and MBP 89–97), and PLP (PLP 80–99). This spreading is highlighted by the progressive increase in the number of antigens targeted on day 28 (Figure 3c) and then on day 67 (Figure 3d).

### Expansion of autoantibody responses against citrullinated epitopes in CIA and EAE

Our microarray experiments characterised the specificity of the autoantibody response to citrullinated proteins and peptides in both CIA (Figure 2) and EAE (Figure 3). Array data from DBA/1 mice immunised with CII emulsified in CFA demonstrated autoantibody reactivity targeting of two citrullinated filaggrin peptides in samples obtained pre-boost on day 21 (Figure 2b). After boosting on day 21 with CII emulsified in IFA, DBA1/J mice developed arthritis about seven days later and array analysis of sera collected on day 31 demonstrated autoantibody responses targeting multiple citrullinated epitopes including cfc1, cfc2, cfc3, cfc4, cfc6 and CCP lin 0139-32 (all citrulline-modified peptides derived from filaggrin) (Figure 2c). Mice with chronic CIA exhibit further expansion of autoantibody reactivity against citrullinated filaggrin peptides (Figure 2d), whereas no reactivity was observed against the native counterparts that were also represented on synovial arrays. For example, in chronic CIA the autoantibody response targeted the citrullinated form of the filaggrin peptide cfc1-3, and showed no reactivity against the corresponding native sequence cfc0 (Figure 2e). ELISA confirmed the presence of anti-CCP antibodies in mice with CIA, while sera from healthy mice did not possess such antibodies (p < 0.001 by Mann-Whitney test).

SJL mice immunixed with PLP 139–151 showed autoantibody reactivity against both native and citrullinated epitopes derived from MBP (Figure 3). These mice contained autoantibodies that were between two and 20-fold more reactive against epitopes containing citrulline as compared with their native counterparts of MBP. In addition, the autoantibody response exclusively targeted the citrullinated epitopes derived from αB-crystallin, the most abundant gene transcript present in early active MS lesions [38]. These experiments also demonstrate development of autoantibody reactivity against citrullinated filaggrin epitopes in EAE (Figures 3c,d), which are believed to represent molecular mimics of the true citrullinated autoantigen in RA.

### Generation of multiple citrullinated polypeptides in CIA joint and EAE brain tissue

**Figure 4:**
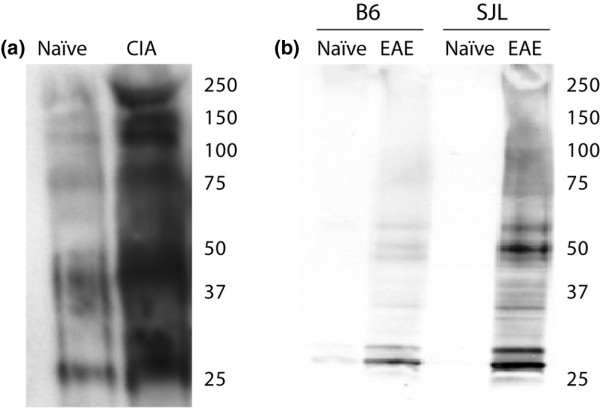
**Immunoblot analysis identifies multiple citrullinated polypeptides in tissue lysates from collagen-induced arthritis (CIA) and experimental autoimmune encephalomyelitis (EAE)**. Lysates were generated from (a) joint tissue harvested from naïve and CIA mice, as well as from (b) brain tissue harvested from naïve and EAE mice. Tissue lysates were separated by SDS-PAGE and subject to immunoblotting with anti-modified citrulline antibodies.

To further investigate the potential targets of anti-citrulline autoantibodies in CIA and EAE, we performed anti-citrulline immunoblotting and mass spectrometry analysis to survey the citrullinated proteins in CIA and EAE. Tissue lysates from CIA joints and EAE brains were separated by SDS-PAGE, and immunoblotting was performed with anti-modified citrulline antibodies. Figure 4 shows the results from representative immunoblots against tissue lysates generated from healthy mice and from mice with CIA (Figure 4a) or EAE (Figure 4b). These blots show an increase in anti-modified-citrulline antibody reactivity to proteins in the CIA joint tissue and EAE brain tissue as compared with the normal control tissues. The reactive polypeptides were excised and identified by mass spectroscopy. Immunoblotting and mass spectrometry analysis of CIA joint tissue lysates identified citrullinated tropomyosin 1 alpha, actin, calgranulin-B and ATP synthase beta chain (Table 1). Immunoblotting and mass spectroscopy analysis of EAE brain tissue lysates identified citrullinated ATP synthase O subunit, peroxisomal membrane protein 20, phosphatidylethanolamine binding protein 1, tyrosine 1-monoxygenase activating protein gamma, malate dehydrogenase 2, glyceraldehyde-3-phosphate dehydrogenase, phosphoglycerate kinase 1, tubulin beta 2c and ATP 5b protein (Table 2).

**Table 1:** Citrullinated peptides detected in collagen-induced arthritis joint tissue

**Protein name**	**Accession number**	**Total score**	**Number of peptides**	**% coverage**	**cit peptide**	**p value**
Tropomyosin 1α	[Genbank: 20522240]	1059	17	45	KLVIIESDLE**(cit)**AEER.A	0.039

Actin	[Genbank: 51316973]	539	10	43	RTTGIVLDSGDGVTHNVPIYEGYALPHAIM**(cit)**L	0.04

Calgranulin-B	[Genbank: 399173]	155	3	58	RSITTIIDTFHQYS**(cit)**K	0.1

ATP synthase β chain	[Genbank: 20455479]	1441	21	66	KGSITSVQAIYVPADDLTDPAPATTFAHLDATTVLS**(cit)**A	0.012
					KSLQDIIAILGMDELSEEDKLTVS**(cit)**A	9.3e-08
					RIMNVIGEPIDE**(cit)**GPIKT	0.47

**Table 2:** Citrullinated peptides detected in experimental autoimmune encephalomyelitis brain tissue

**Protein name**	**Accession number**	**Total score**	**Number of peptides**	**%coverage**	**cit peptide**	**p value**
ATP synthase, H+ transporting, mitochondrial F1 complex, O subunit	[Genbank: 20070412]	205	5	54	KFSPLTANLMNLLAENG**(cit)**L	0.041
					RLASLSEKPPAIDWAYY**(cit)**A	0.016

Peroxisomal membrane protein 20	[Genbank: 6746357]	296	4	50	KATDLLLDDSLVSLFGNR**(cit)**L	0.24

Phosphatidylethanolamine binding protein 1	[Genbank: 84794552]	376	5	52	KLYTLVLTDPDAPS**(cit)**K	0.088
					KVLTPTQVMN**(cit)**PSSISWDGLDPGK.L	0.44
					KGNDISSGTVLSDYVGSGPPSGTGLH**(cit)**Y	7.1e-10

Tyrosine 3-monooxygenase/tryptophan 5-monooxygenase activation protein, ζ	[Genbank: 6756041]	506	14	63	KIETEL**(cit)**DICNDVLSLLEKF	0.0013
					KTAFDEAIAELDTLSEES YKDSTLIMQLL**(cit)**D	2.1e-07

Malate dehydrogenase 2, NAD	[Genbank: 31982186]	650	11	61	KVDFPQDQLATLTG**(cit)**I	0.0025

Glyceraldehyde-3-phosphate dehydrogenase	[Genbank: 55153885]	523	8	41	KLVINGKPTIFQE**(cit)**D	0.096

Phosphoglycerate kinase 1	[Genbank: 146345481]	143	4	18	KDCVGPEVENACANPAAGTVILLENL**(cit)**F	0.066

Tubulin, beta 2c	[Genbank: 13542680]	822	13	46	RSGPFGQIF**(cit)**PDNFVFGQSGAGNNWAKG	0.0056

Atp5b protein	[Genbank: 23272966]	483	15	42	RT**(cit)**EGNDLYHEMIESGVINLKD	0.00035
					KSLQDIIAILGMDELSEEDKLTVS**(cit)**A	9.8e-06

## Discussion

While antibodies against CCPs are considered to be a specific diagnostic marker for RA [39], there is evidence that citrullinated proteins are also generated in tissues affected by other inflammatory diseases including MS. Here we utilised protein arrays to characterise the evolution of autoantibody responses in CIA and EAE. We observe targeting of the native inducing autoantigens and a limited set of citrullinated epitopes in pre-disease and acute disease samples, and expansion of B cell responses to target multiple citrullinated epitopes in both chronic CIA and EAE. The native counterparts of the targeted citrullinated proteins were not targeted by the autoantibody responses in these models.

Our findings extend the results of others who described antibody reactivity to CCP in CIA [14] and the induction of EAE with citrulline-substituted MBP peptides [18]. In CIA we observed development of ACPA responses pre-boosting (Figures 2a,b), which is five to eight days before the onset of arthritis and consistent with the timing of development of anti-CCP antibody responses in CIA reported by others [14]. Following development of arthritis, we observed significant expansion of the ACPA responses in both acute and chronic arthritis (Figure 2) and EAE (Figure 3). It is likely that the pre-arthritis ACPA responses observed in CIA arise due to the local and systemic inflammation that results from immunisation with CFA, and could in part parallel the ACPA responses observed in pre-arthritis samples derived from individuals that subsequently developed RA.

Intramolecular spreading is the expansion of autoantibody responses to target additional epitopes within a polypeptide, while intermolecular spreading is expansion to epitopes on other polypeptides. Our results demonstrate extensive intramolecular and intermolecular spreading of autoantibody responses in both CIA and EAE. It is unclear whether the epitope spreading of ACPA responses observed in acute and chronic CIA and EAE are responsible for the progression of disease or if they arise secondary to joint inflammation. It is possible that joint inflammation results in the generation of citrullinated epitopes, that then act as neoantigens to induce expansion of ACPA responses, that in turn perpetuate arthritis.

To further characterise citrullinated proteins present in the joint and brain tissue under attack in CIA and EAE, we performed immunoblotting and mass spectrometry analysis. We identified multiple previously undescribed citrullinated proteins in joint tissue derived from mice with CIA and brain tissue derived from mice with EAE (Tables 1 and 2). The smear observed in lanes containing the CIA joint protein lysates was most probably due to heavy glycosylation of multiple synovial proteins, and the smearing limited our ability to excise discrete bands for mass spectrometry analysis. Autoantibody targeting of citrullinated antigens in established and advanced, but not acute, disease suggests that as previously hypothesised inflammation of target tissues could result in the aberrant citrullination of multiple proteins, thereby generating neoantigens that provoke autoreactive B cell responses.

Epitope spreading of autoreactive T and B cell responses has been previously described in the EAE model [20,40,41], whereas few researchers have described expansion of autoreactive B cell responses in animal models for RA [42,43]. Our synovial protein array analysis provides further evidence of and insights into epitope spreading in CIA (Figure 2). Our results indicate that epitope spreading in CIA includes development of autoantibody responses targeting citrullinated epitopes at the pre-boost (pre-arthritis) timepoint, and that there is significant expansion of autoantibody responses to target a panel of citrullinated polypeptides in both acute and chronic arthritis. Such anti-citrulline antibody responses could result in more severe arthritis [14].

Epitope spreading of autoantibody responses has been demonstrated to be associated with the development and progression of autoimmune diabetes [44], multiple sclerosis [45] and systemic lupus erythematosus [46], and it remains to be determined if epitope spreading of anti-citrulline autoantibody responses is also associated with the development and/or clinical progression of RA. The purpose of these studies was to characterise the development and evolution of ACPA responses in rodent models of RA and MS, to gain insights into the aetiology and potential pathogenic role of such responses in human RA and MS. Although anti-citrulline responses pre-date clinical arthritis by years in many RA patients, there are several lines of evidence suggesting that such responses can evolve over time in a subset of RA patients: a subset of new-onset RA patients are CCP negative at the time of diagnosis and subsequently develop anti-CCP antibodies [47]; data have suggested that anti-CCP responses evolve both in terms of isotype usage [47] as well as epitope specificity (Robinson laboratory, unpublished data). As a result, the characterisation of the development and expansion of anti-citrulline responses in rodent models of RA and MS could provide insights into a process that may also occur in human RA. It will be important to further characterise the evolution of ACPA responses in human RA, and to determine if the evolution and/or expansion of ACPA responses is associated with the persistence and severity of RA.

Citrullination exists in a variety of tissues and circumstances that are not specifically related to autoimmunity, including normal development of the skin and myelin sheath [4], as well as conditions related to inflammation [48]. As a result, one cannot be sure to what extent the observed effects are specific for autoimmune disease or merely arise secondary to the generation of citrullinated epitopes as a result of inflammation. Further, our results suggest that local or systemic inflammation results in the generation of anti-citrullinated protein responses that react with citrullinated proteins generated in other tissues. This possibility is supported by the reactivity of human RA sera with not only citrullinated proteins generated in inflamed joint tissue but also with citrullinated filaggrin, a protein expressed in stratified epithelium but not in joints [4,6].

It is interesting that many of the citrullinated proteins identified in inflamed CIA joint tissue and EAE brain tissue (Tables 1 and 2) are either structural proteins or enzymes involved in common cellular metabolic pathways. The originally described citrullinated proteins were structural proteins and included fibrinogen, vimentin, keratin, filaggrin and MBP. Citrullination is known to alter the structural properties of keratin and MBP, and thus citrullination may play an important role in modulating the structural properties of such proteins [4]. In our study, immunoblotting and mass spectrometry analysis identified citrullinated tropomyosin 1a and actin in CIA joint tissue, as well as citrullinated tubulin b 2c in EAE brain tissue. To this end, perhaps other structural proteins become accessible to and substrates of PAD in inflamed tissues, such as the joints in CIA and RA, and the myelin sheath in EAE and MS.

The observation of multiple citrullinated metabolic enzymes in inflamed CIA joint and EAE brain tissue is intriguing. Citrullinated enzymes identified included ATP synthase b chain in CIA joint tissue, as well as citrullinated ATP synthase, tyrosine 3 monooxygenase, maltate dehydrogenase, glyceraldehyde-3-phosphate dehydrogenase, phosphoglycerate kinase I and others in EAE brain tissue. Mathis and Benoist demonstrated that autoantibodies targeting the ubiquitously expressed glycolytic enzyme GPI mediate inflammatory arthritis in the K/BxN model [33]. Based on this observation, citrullination of metabolic enzymes could result in such enzymes becoming targets of the ACPA response, and perhaps such responses could result in the formation of immune complexes that would contribute to inflammatory arthritis based on mechanisms analogous to those described by Mathis and Benoist for anti-GPI antibodies. To date, these enzymes have not been described as candidate autoantigens in RA or MS, and their potential role in human disease is unclear.

The synovial and myelin microarrays contained predominantly mouse and/or human versions of the proteins and peptides, depending on their availability from commercial sources, collaborators or the sequence originally used to synthesise peptides (see Additional files 1 and 2). Many of the polypeptide sequences are highly conserved between species, and thus most protein and peptides provide utility for screening of autoantibody reactivity across species. It is likely that use of an array composed entirely of murine proteins and sequences would further accentuate the findings and results we describe herein.

These experiments were performed using an anti-IgM/G secondary antibody, and as a result these experiments did not discriminate IgG versus IgM autoantibody reactivity. IgM and IgG antibodies have fundamentally different properties, including affinity and avidity, ability to activate the complement cascade, and ability to bind Fc receptors and thereby activate effector cells. Further, some IgM antibodies are natural antibodies, while IgG antibodies are produced through adaptive immune responses that result in B cell isotype class switching. The anti-citrullinated fibrinogen antibody that exacerbated anti-collagen antibody-induced arthritis was of the IgM isotype [14], suggesting that anti-citrulline IgM antibodies can exacerbate arthritis in certain rodent models. An important future direction will be characterisation of the isotypes (IgM, IgG and IgG subclasses) of the initial and expanding autoantibody responses in rodent models, as well as human RA and MS.

Autoimmune responses are well established to target both native and linear epitopes present in autoantigens [20]. It is likely that autoantibody targeting of native CII epitopes is particularly important in CIA, as evidenced by the observation that CIA is only inducible with whole CII or certain large fragments of CII. CIA is not inducible with peptides derived from CII, which differentiates it from EAE in which short peptides derived from multiple myelin antigens induce disease in multiple mouse strains. Although low level antibody responses are observed against several CII peptides (CII 194–213, CII 224–243, CII 354–373, CII 444–463 and others), the strongest anti-CII response is observed against native CII and probably reflects the importance of targeting native CII epitopes in the pathogenesis of CIA.

## Conclusion

ACPAs develop pre-arthritis in CIA and early EAE. Autoimmune inflammation in both the joint and brain is associated with the generation of citrullinated polypeptides that probably further induce epitope spreading of ACPA responses. The expansion of autoantibody responses to neoantigens, including citrullinated epitopes generated in inflamed tissues, may expand and effectively boost ACPA responses that may contribute to more severe and chronic disease in CIA and perhaps EAE. Further investigation is needed to better understand why anti-citrulline responses develop in inflammatory and autoimmune states, and in humans why they appear to be specific to RA.

## Abbreviations

ACPA: anti-citrullinated protein antibody; BCA: bicinchoninic acid; BSA: bovine serum albumin; CII: collagen type II; CCP: cyclic citrullinated peptide; CFA: complete Freund's adjuvant; CIA: collagen induced arthritis; dfu: digital fluorescence units; EAE: experimental autoimmune encephalomyelitis; FCS: fetal calf serum; GPI: glucose-6-phosphate isomerase; HPLC: high performance liquid chromatography; HRP: horse radish peroxidase; IEF: isoelectric focusing; MBP: myelin basic protein; MOG: myelin-associated oligodendrocyte; MS: multiple sclerosis; PAD: peptidyl arginine deiminase; PBS: phosphate buffered saline; PLP: proteolipid protein; RA: rheumatoid arthritis; SDS-PAGE: sodium dodecyl sulfate polyacrylamide gel electrophoresis.

## Competing interests

The authors declare that they have no competing interests.

## Authors' contributions

BAK participated in the design of the experiments, performed array and ELISA studies, conducted statistical analyses and participated in the writing of the manuscript. PPH and JLK carried out animal studies. OS carried out immunoblot and mass spectrometry experiments. BHT performed array experiments. LS participated in the design of experiments. WHR conceived the study idea and participated in its design, data analysis and the writing of the manuscript.

## Supplementary Material

Additional file 1 File containing a table that lists the proteins and peptides included on myelin arrays. Click here for file

Additional file 2 File containing a table that lists the proteins and peptides included on synovial arrays. Click here for file
